# Parasite-microbe-host interactions and cancer risk

**DOI:** 10.1371/journal.ppat.1007912

**Published:** 2019-08-15

**Authors:** Nolwenn M. Dheilly, Paul W. Ewald, Paul J. Brindley, Raina N. Fichorova, Frédéric Thomas

**Affiliations:** 1 School of Marine and Atmospheric Sciences, Stony Brook University, Stony Brook, New York, United States of America; 2 Department of Biology, University of Louisville, Louisville, Kentucky, United States of America; 3 Department of Microbiology, Immunology and Tropical Medicine and Research Center for Neglected Diseases of Poverty, School of Medicine and Health Sciences, George Washington University, Washington DC, United States of America; 4 Department of Obstetrics, Gynecology and Reproductive Biology, Brigham and Women’s Hospital, Harvard Medical School, Boston, Massachusetts, United States of America; 5 CREEC (UMR CNRS/IRD/UM1/UM2 5290), BP, France; University of Wisconsin Medical School, UNITED STATES

## Can natural selection act on parasites to compromise barriers to cancer?

Characterizing the factors that disrupt the cellular barriers to cancer (e.g., cell-cycle arrest, apoptosis, repression of telomerase, cell adhesion, and asymmetric cell division) and are essential to oncogenesis is necessary to identify targets for therapeutic interventions [1]. Exacerbating causes can contribute to cancer by compromising host restraints on cancer rather than breaking barriers; examples of such exacerbating causes are factors that drive angiogenesis, or increased proliferation during pro-inflammatory responses [1]. All viruses that are recognized by the International Agency for Research on Cancer (IARC) as Group 1 carcinogens, namely human papillomaviruses (HPV), Hepatitis B and C viruses (HBV and HCV), Human Herpes Virus type 8 (HHV-8), and Human T-cell lymphotropic virus type 1 (HTLV-1) [2], break barriers to cancer and therefore generate essential causes of their associated cancers [1]. These may be the result of natural selection. For example, from an evolutionary point of view, it is probably advantageous to a virus that its host cell resists cell death, evades the immune system, and proliferates. Other intracellular organisms (bacteria and unicellular eukaryotes) could similarly benefit from altering the cellular mechanisms that prevent oncogenesis. Indeed, the ability of intracellular bacteria and protozoan parasites to block apoptosis is now broadly recognized [3,4]. Increasing evidence implicates bacteria (certain strains of *Escherichia coli*, *Fusobacterium nucleatum*, *Salmonella* Typhi, *Chlamydia trachomatis*, and a range of *Mycoplasma*) and protists (*Cryptosporidum parvum*, *Trichomonas vaginalis*, *Trypanosoma cruzi*, *Toxoplasma gondii)* in cancer development [2, 5–26].

For extracellular parasites, and in particular helminths, the evolutionary path that could lead them to break the cellular barriers to cancer is more difficult. At present, three multicellular parasites, the trematodes *Schistosoma haematobium*, *Opisthorchis viverrini*, and *Clonorchis sinensis* are recognized as Group 1 carcinogens by IARC, contributing 0.4% to human cancer [2]. However, increased risk of cancer is associated with increasing numbers of other parasites, e.g., species of *Echinococcus*, *Strongyloides*, *Fasciola*, *Heterakis*, *Platynosomum*, and *Trichuris* [27] and is likely broadly underestimated due to the asymptomatic/subclinical nature of some of these infections, wide occurrence among healthcare underserved communities, and long latency between initial infection or exposure and clinical manifestation of cancer. The latest advances in microbiology suggest a new paradigm—multi-microbial factors could explain why and how some parasites breach host barriers to cancer. Herein, we review the literature indicating that microbes can contribute in an essential sense to oncogenesis through their interaction with the host and with parasites.

## High prevalence of microbes with oncogenic potential in asymptomatic populations

The composition of the microbiome is thought to result from complex co-evolutionary mechanisms among hosts and microbes. Antagonistic pleiotropy refers to genes that are beneficial early in life, and improve fitness, but become detrimental later in life [28]. Recent studies suggest that microbes and microbial communities can have similar effects: microbiome composition induces a life-history trade-off between life span and reproduction in flies [29]. Thus, natural selection will favor establishment of microbes that are either beneficial to the host or present a low cost of infection early in life, even if these microbes promote oncogenesis later in life. Indeed, the human microbiome includes known and often highly prevalent oncogenic microbes, such as Epstein Barr Virus (EBV), that infect 90% of the human population [30], and many members of the gut microbiota are associated with cancer [6]. Only a small fraction of the population carrying oncogenic microbes develops cancer, suggesting that cofactors that exacerbate the susceptibility to cancer are necessary. Other microbes that are not identified as oncogenic may show high prevalence and present as asymptomatic infection, but may enact essential causes of cancer. Microbial communities previously regarded as commensal may have diverse roles in oncogenesis during the long asymptomatic/subclinical period preceding clinical diagnosis of cancer.

## Parasites can modulate the oncogenicity of host-associated microbes

Coinfection by multiple parasites is common in the wild. If we consider that any host-associated microbe can move along the parasitism-mutualism spectrum in a context-dependent manner, coinfection is the norm. Viruses, bacteria, archaea, and eukaryotic parasites have coinhabited the same host lineages for hundreds of millions of years and can either directly interact when they inhabit the same host tissue or indirectly interact via modulation of the host immune system. These interactions can influence tumor development and progression.

A widely appreciated example is the role of *Plasmodium falciparum* as an indirect risk factor for Burkitt lymphoma, a monoclonal B cells cancer for which EBV infection is generally considered essential [31]. Recent studies have clarified the mechanisms by which *P*. *falciparum* contributes to oncogenesis. The immunosuppression associated with *P*. *falciparum* malaria leads to an increase in EBV-infected B cells in the germinal center, which dysregulates activation-induced cytidine deaminase expression, leading to DNA damages, including *c-myc* translocation that should lead to cell apoptosis, but EBV rescues the infected cell by inhibiting apoptosis, effectively leading to Burkitt lymphoma [32–34].

Similarly, infection with *Strongyloides stercoralis*, a common parasitic nematode, shortens the delay between HTLV-1 infection and the occurrence of T-cell leukemia [35, 36]. *S*. *stercoralis* benefits HTLV-1 with a higher proviral load in individuals infected by the roundworm, due to the proliferative expansion of HTLV-1–infected cells [37]. By promoting cell proliferation, *S*. *stercolis* is an exacerbating cause of cancer. Infection with HTLV-1 results in a suppressed immune response against helminths and in the reduced efficacy of antiparasitic drugs, which lead to higher prevalence of *S*. *stercoralis* infection in HTLV-1–infected individuals [38].

Evidence is accumulating that members of the gut, oral, and vaginal microbiomes could initiate or influence the progression of oncogenesis by different processes, including the induction of a chronic inflammatory state or immune response, altering stem cell dynamics, the biosynthesis of toxic and genotoxic metabolites, and affecting host metabolism [6, 39, 40]. Many parasites significantly alter their host microbiome composition [41]. Outcomes would therefore depend on the individual’s microbiome composition at the time of infection, which would render the association between parasite infection and cancer difficult to resolve.

A positive association between the highly prevalent sexually transmitted protozoan parasite *T*. *vaginalis* and cervical neoplasia in women and prostate cancer in men has been reported [13–18, 42]. *T*. *vaginalis* infection significantly affects the vaginal microbiome with a shift from a lactobacillus-dominated microbiome to a community of bacteria responsible for the widely spread syndrome of bacterial vaginosis [43]. Metabolites released by the parasite, e.g., indole, support the survival of intracellular sexually transmitted bacteria such as *C*. *trachomatis*, which has been independently associated with cancer [44]. Given the positive association between bacterial vaginosis and cervical precancerous lesions [45], studies are needed to clarify the role of the microbiota as a cofactor, or essential factor, for *T*. *vaginalis*–associated cancer.

*S*. *haematobium* is the causative agent of urogenital schistosomiasis (UGS). This trematode parasite is endemic in 76 countries in Africa and the Middle East, but it can also be found in Europe [46, 47]. UGS is a major risk factor for squamous cell carcinoma of the urinary bladder [48, 49]. Early studies have found that UGS promotes bacterial coinfection and is associated with a high concentration of *N*-nitroso compounds in the urine, suggesting that infection favors nitrate-reducing bacteria that produce the cancer-inducing nitrosamines [50–52]. More recent studies of the microbiome of noninfected and infected patients also revealed marked differences [53, 54]. In addition, schistosomes can directly interact with bacteria, and *Salmonella* is known to routinely attach to a range of species of schistosomes [55]. HPV, EBV, and BK polyomavirus (BKV) have been found in a minority of bladder cancers by some investigators, although not by others [56]. Given the potential for some of these viruses and bacteria to cause cancer, studies are needed to test their roles as causative agents for urinary bladder cancer associated with UGS.

## Parasites can transmit pro-inflammatory or oncogenic microbes

There are numerous compelling examples of parasites that carry microbes that participate in the infectious process, notably the well-documented *Wolbachia*-filarial nematodes system [57, 58]. There is even evidence that parasites have received genes from prokaryotic symbionts via horizontal gene transfers, including numerous *Wolbachia* genes in symbiont-free filarial nematodes [59], the thymidine kinase of *C*. *parvum* [60], and the *N*-acetylneuraminate lyase of *T*. *vaginalis* [61], further demonstrating the selective advantage that microbial symbionts confer to their parasitic hosts. Microbial symbionts of parasites can be transmitted to the host and be responsible for inflammatory-associated pathogenesis [62, 63]. In addition, the release of *Wolbachia* from filarial nematodes and of *Trichomonavirus* (TVV) from *T*. *vaginalis* upon parasite death have significant adverse effects that impair treatment efficiency [62, 63]. Similarly, relapse of *Salmonella* infection can occur in the absence of antischistosomal treatment, probably because of the antibiotic resistance of *Salmonella* attached to the blood fluke [64, 65]. Could the ability of parasite-associated microbes to infect host cells, and their role in inflammation, be responsible for oncogenesis, in lieu of their parasitic host?

Infection with the liver fluke *O*. *viverrini* is recognized as a definitive cause of cancer, as infection often leads to cholangiocarcinoma (bile duct cancer). The factors that lead to cancer development have not yet been clearly identified [66]. One intriguing hypothesis is the potential for *O*. *viverrini* to serve as a vector of the oncogenic bacterium *Helicobacter pylori* and other bacteria into the biliary tree, triggering the malignant transformation of cholangiocytes [67]. Indeed, *H*. *pylori* was found within the gut of *O*. *viverrini* [68], and coinfection is associated with higher expression of pro-inflammatory cytokines and more severe hepatobiliary morbidity, suggesting that the bacteria contributes to opisthorchiasis-associated cholangiocarcinoma [69, 70]. Sequencing of prokaryotic 16S genes from *O*. *viverrini* revealed the presence of diverse bacteria, including *Bordetella*, *Brochothrix*, *Burkholderia*, *Leminorella*, *Pseudomonas*, *Serratia*, and *Sphingomonas* [71]. The role of other microbes, such as viruses, in the disease cannot be excluded given the ability of *O*. *viverrini* to convey bacteria to the biliary tract.

It is now well recognized that *T*. *vaginalis* hosts a complex core microbiome composed of double-stranded RNA (dsRNA) endobiont viruses of the genus TVV and eubacterial *Mycoplasma* species that substantially increase *T*. *vaginalis* pathogenicity by up-regulating pro-inflammatory responses [72]. Transmission of *M*. *hominis*, a member of the *T*. *vaginalis* microbiome, is associated with malignant transformation and genome instability that promote prostate cancer development in men and skew the adaptive immune response towards a T-helper 17 cell phenotype, thus creating a favorable environment for tumor development [19, 72–75]. TVV can trigger endosomal TLR3/TRIF-dependent pathways, which means that it can penetrate human cells and could also cause oncogenic damage [62]. Both TVV and *Mycoplasma* can resist clearance by the host and antimicrobial therapy, which explains adverse effects of metronidazole treatment [62, 72]. Should the bacterial or viral symbionts of *T*. *vaginalis* induce cancer associated with trichomoniasis, novel therapy could be developed to block malignant transformation in both men and women.

For most parasites, the presence of microbes residing in or directly associated with parasites has not been investigated, and when microbes have been observed, their contribution to oncogenesis has not been assessed. For example, and focusing on parasites listed above that have been linked to cancer prevalence, virus-like particles have been observed in *T*. *cruzi* [76], a dsRNA virus has been found in *Cryptosporidium* and virus load correlates with parasite fecundity [8, 77], *Heterakis gallinarum* is a vector of the pathogenic bacterium *Histomonas meleagridis* [78, 79], *Trichuris muris* hosts a complex bacterial microbiome [80], *Schistosoma mansoni* might be a vector of HCV [81], and genome sequencing of *Fasciola hepatica* revealed the presence of the endobacterium, *Neorickettsia* [82]. In view of these examples, the general lack of information highlights the value of a comprehensive characterization of the viral and bacterial communities associated with parasites [83], and of epidemiological studies that assess the presence of parasites, the prevalence of known microbes, and the transmission to the host, to identify prospective microbial cofactors of oncogenesis.

## Conclusions

**Fig 1:**
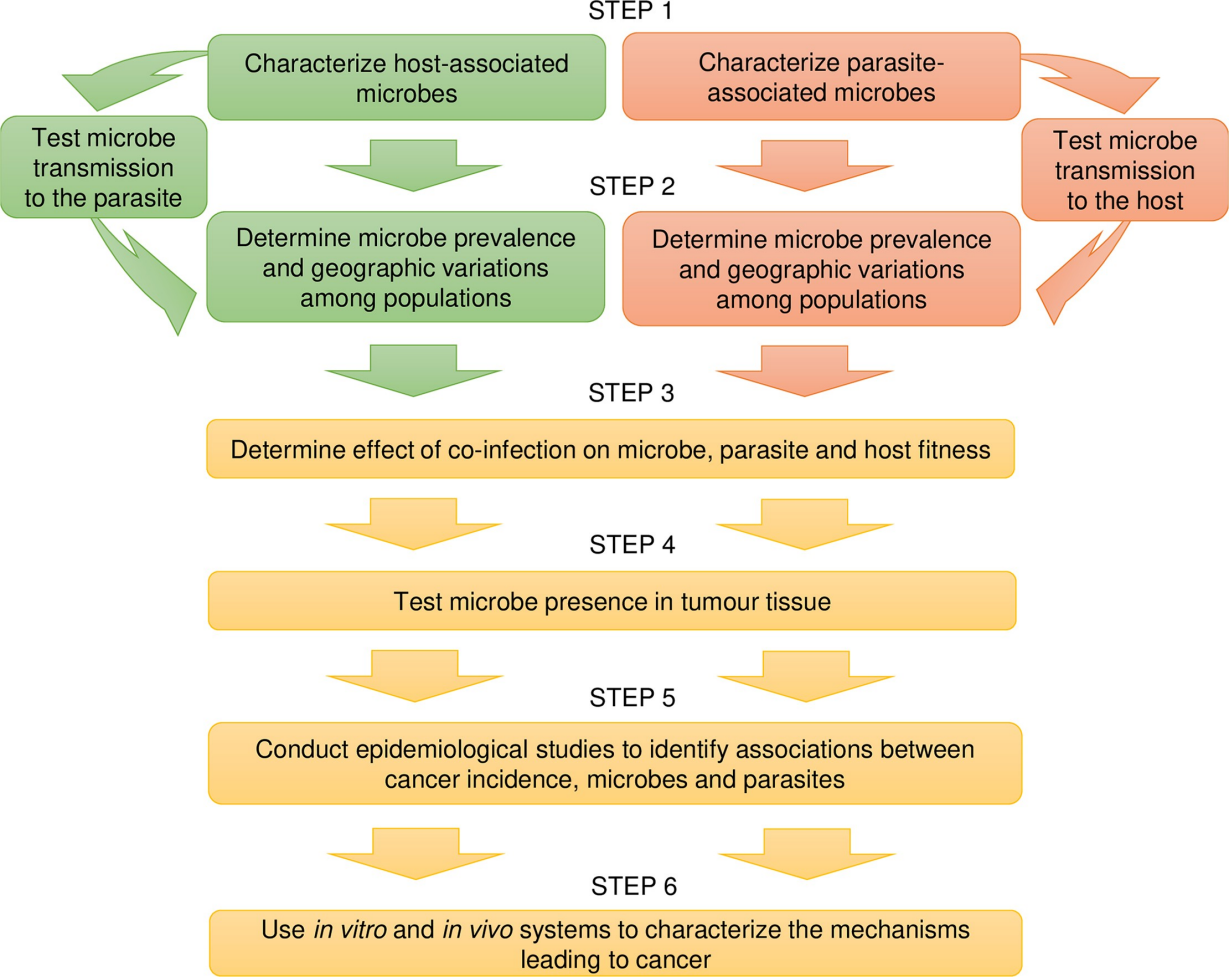
Flowchart of experimental and epidemiological studies that can be undertaken to assess the role of microbes in oncogenesis.

Interindividual variations in microbial communities associated with the host or with the parasite at the time of infection could explain apparently contradictory results in parasite association with cancer among studies due to variations in microbe prevalence among populations. Variations in microbial communities could also explain why some patients, but not others, develop cancer. The task of identifying the contribution of parasites and microbes to cancer can appear overwhelming, but causal inference is feasible with a combination of experimental and epidemiological studies (Fig 1). Following the systematic characterization of microbes associated with parasites, as proposed by the Parasite Microbiome Project [83], and by leveraging the findings from other projects such as the Human Microbiome project [84, 85], epidemiological and clinical studies of cancer could investigate the potential for coinfection by different parasites and microbes, and investigate their interacting effects. Testing for the role of microbes in cancer attributed to parasites has the potential to propel the field forward by revealing cofactors that contribute to the development of precancerous lesions and to the transition from benign to malignant cancer. The presence of newly identified microbes in archived cancer tissues should also be tested to assess their potential role. The payoff for identifying microbial factors that contribute to oncogenesis would be self-evident and compelling with respect to new leads for clinical intervention and prevention. In particular, if a virus plays a causal role or exacerbates cancer progression, vaccine development would be justified, as demonstrated by the protection against both infection and infection-associated cancers delivered by the acclaimed HBV and HPV vaccines [86, 87].
